# Relation between leukocyte count, adiposity, and cardiorespiratory fitness in pubertal adolescents

**DOI:** 10.1590/S1679-45082014AO3214

**Published:** 2014

**Authors:** Thiago Ricardo dos Santos Tenório, Breno Quintella Farah, Raphael Mendes Ritti-Dias, João Paulo Botero, Daniel Calado Brito, Patrícia Muniz Mendes Freire de Moura, Wagner Luiz do Prado

**Affiliations:** 1 Universidade de Pernambuco, Recife, PE, Brazil.; 2 Universidade Federal de São Paulo, Santos, SP, Brazil.

**Keywords:** Obesity, Adolescent, Leukocyte count, Adiposity, Physical fitness, Inflammation

## Abstract

**Objective:**

To compare the total and differential leukocyte count in obese and normal-weight adolescents, and to verify their possible relations with cardiorespiratory fitness and adiposity indicators.

**Methods:**

A cross-sectional study conducted with 139 adolescents (107 obese and 32 normal weight) aged between 13 and 18 years. Cardiorespiratory fitness was determined by direct gas analysis during an incremental treadmill test. Total leukocytes and subsets were estimated by flow cytometry. Body composition was assessed by dual-energy X-ray absorptiometry. The *t*-test for independent samples was used for comparison between groups. The relation between leukocytes, cardiorespiratory fitness and adiposity indicators was verified by Pearson’s correlation and multiple linear regression (adjusted for age and body mass index) tests.

**Results:**

Obese adolescents had higher leukocyte (8.12±2.36u/L x 10^3^; p=0.001), neutrophil (4.33±1.86u/L x 10^3^; p=0.002), and monocyte (0.70±0.22u/L x 10^3^; p=0.002) counts compared to the levels of normal weight subjects. After the necessary adjustments, cardiorespiratory fitness had a negative association with leukocytes, neutrophils, and monocytes in boys.

**Conclusion:**

Obese adolescents had higher total and differential leucocyte count when compared to normal weight individuals. We also observed a weak positive association between adiposity and total leukocyte, monocyte, and neutrophil counts, and in boys, a negative association between cardiorespiratory fitness and total count of leukocytes, monocytes, and neutrophils.

## INTRODUCTION

Obesity has become a global epidemic, affecting about 500 million adults.^(1)^ Excessive adiposity is associated with various cardiometabolic risk factors (such as glucose intolerance, hypertriglyceridemia, hypercholesterolemia, and increased systolic blood pressure),^(2)^ which lead to the probability of early death.^(3)^ Additionally, evidence shows that, in obesity, there is a significant increase in the production of inflammatory markers and that a large part of these cardiometabolic complications could be related to this chronic systemic inflammatory status in obese individuals.^(4,5)^

Leukocyte count with its subpopulations is a clinical marker of inflammatory processes^(6,7) ^related to cardiometabolic disorders involved in the development of cardiovascular diseases,^(8)^ especially in overweight individuals.^(9)^

On the other hand, cardiorespiratory fitness has been considered a protective factor against health problems, both in adults^(10)^ and in children and adolescents.^(11,12)^ Prior studies showed an independent negative relation between levels of leukocytes and cardiorespiratory fitness in men^(13)^ and women.^(14)^ However, despite the fact that cardiometabolic disorders associated with obesity can be triggered during early childhood,^(15)^ little is known about this association in childhood and adolescence.

## OBJECTIVE

To compare the total and differential count of leukocytes in obese and normal-weight adolescents, and to investigate possible associations with cardiorespiratory fitness and adiposity.

## METHODS

A cross-sectional study carried out at the *Escola Superior de Educação Física da Universidade de Pernambuco,* with 139 adolescents (15.21±1.51 years), in which 107 were obese (63 girls) recruited for the adolescent obesity treatment of the university and 32 normal-weight adolescents (22 girls). The study period was between January and April, 2013.

The adolescents met the following inclusion criteria: age between 13 and 18 years and maturation stage between 3 and 4, as per Tanner’s criteria,^(16)^ absence of arterial hypertension and/or other metabolic diseases (self-reported or identified by the endocrinologist in charge), body mass index (BMI) above the 95^th^ percentile (obese) and between the 25^th^ and 85^th^ percentiles (normal weight).^(17)^ Adolescents who reported excessive use of alcohol, tobacco, or continuous use of anti-inflammatories and/or antihistamines were excluded from the sample.

The study was conducted in accordance with the principles of the Declaration of Helsinki, and was formally approved by the Ethics Committee of the *Universidade de Pernambuco* (CAAE: 15796113.9.0000.5207). The Informed Consent of the parents or legal guardians and the participant’s favorable opinion were obtained after a detailed explanation of the procedures of the entire study protocol.

### Anthropometry and body composition

Height and body mass were measured by means of a stadiometer and calibrated scales (Welmy^®^) with a precision of 0.1cm and 0.1kg, respectively. At the time, the subjects were wearing light clothing and no shoes. The BMI was calculated by dividing the body weight (kg) by the squared height (m^2^). Body composition was determined by dual-energy X-ray absorptiometry (DXA) (model QDR HOLOGIC WI).

### Cardiorespiratory fitness

Peak oxygen uptake (VO_2peak_) was used to determined cardiorespiratory fitness. VO_2 _was analyzed directly in an open circuit respiratory metabolic system (Quark PFT, Cosmed, Italy) during continuous incremental treadmill testing (Cosmed T200, Cosmed, Italy). Before each test, the equipment was calibrated for reference gas composition (O_2_=12.2% and CO_2_=4.8%; White Martins), following the manufacturer’s recommendations. The initial load was set at 4km/h (warm-up for 3 minutes) and elevated to 1km/h each minute, with inclination kept constant at 1%. The interruption criteria of the test were voluntary fatigue, Borg’s scale, and gas exchange ratio of more than 18 and 1.15, respectively. The highest value of VO_2_ obtained before the test was interrupted was considered the VO_2peak_.

### Blood analyses

Blood samples were collected from the peripheral vein of the forearm, with tubes containing anticoagulant (EDTA), after a night of fasting (12 hours). Total and differential leukocyte count (neutrophils, monocytes, and lymphocytes) was determined by means of the fluorescent flow cytometry (Sysmex XE 2100^®^).

### Statistical analysis

To analyze normality and homogeneity of data distribution, the Kolmogorov-Smirnov and Levene tests, respectively, were used. To compare the concentration of leukocytes and subsets between obese and normal-weight adolescents, Student’s *t*-test or Mann-Whitney’s (non-parametric) test was used for independent samples. Spearman’s correlation was used to analyze the relation between body composition, cardiorespiratory fitness (VO_2peak_), and leukocyte concentrations. Multiple linear regressions adjusted for age and BMI were used to analyze the independent relation between cardiorespiratory fitness and leukocyte count (with subpopulations). All the statistical procedures were performed using the Statistical Package for the Social Sciences program (SPSS Inc., Chicago, United States), version 20.0. The level of statistical significance was set at p<0.05.

## RESULTS

Table 1 shows the anthropometric parameters, body composition, and cardiorespiratory fitness by group. The obese adolescents had a lower age (p<0.001) and VO_2peak_ (p<0.001); on the other hand, they had greater BMI, fat percentage, fat mass, and lean mass (p<0.001, for all of them) in comparison to normal weight adolescents.

**Table 1:** Anthropometric, body composition, and cardiorespiratory fitness characteristics

Variables	Obese (n=107)	Normal weight (n=32)	p value
	X±SD/MED(IA)	X±SD/MED(IA)	
Age (years)	14.28 (2.24)	16.78 (1.27)	0.001*
Body mass (kg)	93.73±13.51	56.32±7.30	0.001**
BMI (kg/m^2^)	34.28±4.06	20.79±1.86	0.001**
Fat (%)	50.41±4.85	29.16±8.06	0.001**
Fat mass (kg)	46.57 (13.12)	15.15 (12.50)	0.001*
Lean mass (kg)	44.26±7.27	38.20±8.16	0.001**
VO_2peak_ (ml.Kg/min^1^)	24.90±4.16	33.60±5.75	0.001**

X: mean; SD: standard deviation; MED: median; IA: interquartile amplitude; BMI: body mass index; VO_2peak_: peak oxygen uptake.* Mann-Whitney U Test; ** *t*-test for independent samples.

**Figure 1:**
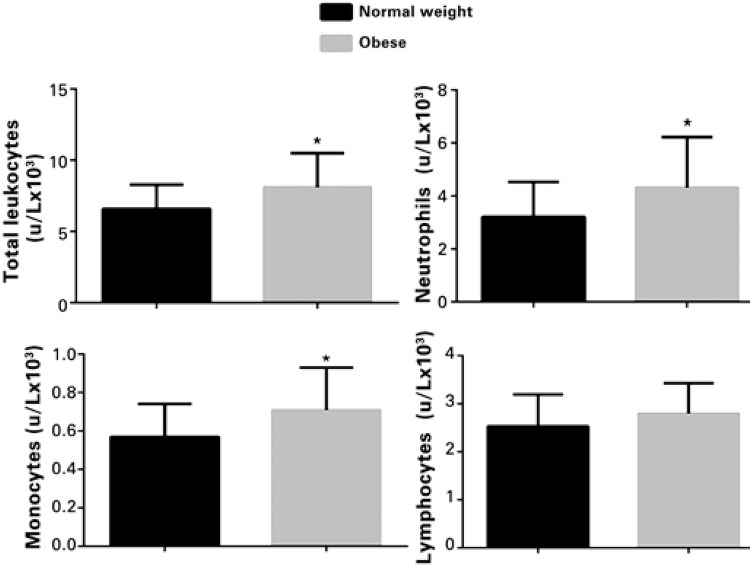
Comparison of leukocyte and subpopulation counts in obese and eutrophic adolescents. *p<0.05

Obese adolescents had higher total leukocyte (8.12±2.36 x 10^3^u/L; p=0.036), neutrophil (4.33±1.86 x 10^3^u/L; p=0.002), and monocyte (0.70±0.22 x 10^3^u/L; p=0.001) counts compared to those who were normal weight. There was no difference between the groups as to lymphocyte subpopulation counts (p=0.120) (Figure 1).

There was a significant correlation between the total leucocyte count and the percentage of fat (r=0.22), fat mass – in kilograms (r=0.25), BMI (r=0.22), and VO_2peak_ (r=-0.22). Also observed were significant correlations between neutrophils and percentage de fat (r=0.27), fat mass – in kilograms (r=0.24), and VO_2peak_ (r=-0.25); between monocytes and fat mass − in kilograms (r=0.19) and BMI (r=0.18); and among the neutrophils/lymphocytes ratio and percentage of fat (r=0.22) and fat mass − in kilograms (r=0.18) (Table 2).

**Table 2:** Correlation between leukocyte count and subsets with anthropometric parameters of body composition and cardiorespiratory fitness (n=139)

Variables	%G	FM	LM	BMI	VO_**2peak**_
Total leukocytes (u/L x 10^3^)	0.27*	0.25*	0.08	0.22*	-0.22*
Neutrophils (u/L x 10^3^)	0.27*	0.24*	0.05	0.21*	-0.25*
Monocytes (u/L x 10^3^)	0.17	0.19*	0.13	0.18*	-0.10
Lymphocytes (u/L x 10^3^)	0.13	0.15	0.11	0.12	-0.03
Neutrophil/lymphocyte ratio	0.22*	0.18*	-0.01	0.17	-0.24

%G: percentage of fat; FM: fat mass; LM: lean mass; BMI: body mass index; VO_2peak: _Peak oxygen uptake. Spearman’s correlation. *p< 0.05.

Table 3 demonstrates the relations among total leukocytes, neutrophils, and monocytes with cardiorespiratory fitness, when adjustments are made for age and BMI. In boys, total leukocytes, neutrophils, and monocytes proved to have a negative association with cardiorespiratory fitness. Among the girls, this type of relation was not seen.

**Table 3:** Association among cardiorespiratory fitness, total leukocytes, neutrophils, and monocytes (n=139)

Dependent variables	Cardiorespiratory fitness (VO_**2peak**_)		
	β	SE	p value
Boys			
Total leukocytes (u/Lx10^3^)	-0.534	0.095	0.013
Neutrophils (u/Lx10^3^)	-0.545	0.067	0.012
Monocytes (u/Lx10^3^)	-0.019	0.008	0.042
Girls			
Total leukocytes (u/Lx10^3^)	-0.003	0.093	0.977
Neutrophils (u/Lx10^3^)	0.013	0.078	0.868
Monocytes (u/Lx10^3^)	-0.002	0.008	0.998

Multiple linear regression: model adjusted for age and body mass index. β: regression coefficient; SE: standard error.

## DISCUSSION

These were the primary findings of this study: obese adolescents presented with higher total leukocytes and subpopulation counts (neutrophils and monocytes) when compared to their normal weight peers; there was a positive relation between adiposity and total leukocytes, monocytes, and neutrophils; there was also a negative association between cardiorespiratory fitness and total leukocytes, monocytes, and neutrophils, only for the boys, regardless of BMI and age.

Prior studies reported a chronic low-grade state of inflammation in obese adolescents.^(18,19)^ The highest leukocyte counts observed in obese adolescents in this study were similar to results in adults,^(9)^ children^(20)^ and adolescents.^(21)^ In fact, the positive correlations found between total counts of leukocytes ad subpopulations (neutrophils and monocytes), even with weak intensities (r=0.18 to r=0.27) already shown in prior studies,^(6,7)^ indicated that, in part, total leukocyte, monocyte, and neutrophil counts suffer the influence of adiposity, with a reflex, globally, on the inflammatory profile of adolescents.

Another point refers to the significant difference between the ages of the obese and normal weight adolescents. However, all the adolescents obtained a nutritional status classification by means of percentile curves^(17)^ that took into consideration the individual age of each teen. Additionally, the regression analyses were corrected by age, in order to isolate any influence of this variable on the results found.

In adults, there are descriptions that a high leukocyte count is an independent risk factor for the development of cardiovascular diseases.^(22)^ The mechanisms for this effect are not yet totally clear, but it is probably related to the release of free radicals, procoagulant molecules, and proteolytic enzymes by neutrophils and monocytes, which could accelerate the process of formation of the atherosclerotic plaque.^(20)^ Additionally, monocytes secrete the tumor necrosis factor alpha (TNF-α), a cytokine that is related to insulin resistance.^(23)^

Moreover, neutrophils and monocytes have been associated with coronary artery disease.^(24)^ Our results suggest that the effects of these harmful processes may begin earlier and earlier, which, on the other hand, might indicate that obesity in adolescence may trigger the appearance of coronary artery disease.

Another important finding of this study was a negative correlation between the VO_2peak_ and total leukocyte and neutrophil counts. Although the VO_2peak_ is considered a marker for physical fitness, it is also important to point out that such a parameter reflects the function of the cardiopulmonary system, and is considered a parameter of protection against risk factors for atherosclerosis.^(25) ^Michishita et al.,^(14)^ in a study conducted in women with excess weight, observed a negative correlation between monocytes and VO_2peak_. In this way, it is believed that the elevation in cardiorespiratory fitness of obese adolescents may influence the decrease in production of inflammatory markers, providing a greater anti-inflammatory state, which is important in the prevention of cardiovascular events.

Interestingly, the association between cardiorespiratory fitness and leukocyte and subpopulation counts, after adjustments for BMI and age, was observed only in girls. Such a result is in accordance with that of prior studies in populations of more advanced ages.^(26,27)^ The reason for this difference between genders is not totally understood, but the lower level of physical activity, generally observed in girls, is a potential factor.^(28)^ Additionally, there is a close relationship between sexual steroids, inflammation, and body fat distribution in women since due to the menstrual cycle, the circulating levels of inflammatory markers may vary significantly.^(29)^

In this sense, leukocyte adhesion to the endothelium, especially monocytes and neutrophils, as well as the migration of these cells to the walls of blood vessels are characteristics involved in various phases of atherosclerosis.^(6)^ Adamopoulos et al.^(30)^ demonstrated that the increase in VO_2peak_ after a period of aerobic training was effective in inhibiting monocyte infiltration in the vessel wall. Thus, higher levels of VO_2peak_ afford protective cardiovascular effects, inhibiting inflammatory processes.

The primary limitations of the study were the isolated analysis of the leukocyte count, since the evaluation of its function and of its activation could provide important information; the non-control of menstrual periods of the adolescents; and finally, the non-evaluation of the influence of the fat distribution pattern.

On the other hand, it is important to point out that in the present study, the VO_2peak_ was determined by direct gas analysis, and body composition was assessed by means of dual-energy X-ray absorptiometry (DXA). Such methods reinforce the results of this study, since they deal with more precise and accurate techniques, especially related to the study population.

## CONCLUSION

Higher levels of leukocytes (monocytes and neutrophils) were observed in obese adolescents in comparison with those who were eutrophic. This profile suggested a chronic pro-inflammatory status in these obese teens, which might be related to the excessive adiposity and decreased cardiorespiratory fitness. Such data highlight the eminent need for the development of interventions in this population, seeking not only weight control, but also improvements in cardiorespiratory fitness.

## References

[1] Finucane MM, Stevens GA, Cowan MJ, Danaei G, Lin JK, Paciorek CJ, Singh GM, Gutierrez HR, Lu Y, Bahalim AN, Farzadfar F, Riley LM, Ezzati M (2011). Global Burden of Metabolic Risk Factors of Chronic Diseases Collaborating Group (Body Mass Index). National, regional, and global trends in body-mass index since 1980: systematic analysis of health examination surveys and epidemiological studies with 960 country-years and 9·1 million participants. Lancet.

[2] Abbasi F, Blasey C, Reaven GM (2013). Cardiometabolic risk factors and obesity: does it matter whether BMI, or waist circumference is the index of obesity. Am J Clin Nutr.

[3] Franks PW, Hanson RL, Knowler WC, Sievers ML, Bennett PH, Looker HC (2010). Childhood obesity, other cardiovascular risk factors and premature death. N Engl J Med.

[4] Gregor MF, Hotamisligil GS (2011). Inflammatory mechanisms in obesity. Annu Rev Immunol.

[5] Rocha VZ, Folco EJ (2011). Inflammatory concepts of obesity. Int J Inflam.

[6] Farhangi MA, Keshavarz AS, Eshraghian M, Ostadrahimi A, Sabbor-Yaraghi AA (2013). White blood cell count in women: relation to inflammatory biomarkers, haematological profiles, visceral adiposity, and other cardiovascular risk factors. J Health Popul Nutr.

[7] Ganguli D, Das N, Saha I, Sanapala KR, Chaudhuri D, Gosh S (2011). Association between inflammatory markes and cardiovascular risk factors in women from Kolkata, W.B, India. Arq Bras Cardiol.

[8] Tatsukawa Y, Hsu WL, Yamada M, Cologne JB, Suzuki G, Yamamoto H (2008). White blood cell count, especially neutrophil count, as a predictor of hypertension in a Japanese population. Hypertens Res.

[9] Dixon JB, O’Brien PE (2006). Obesity and the white blood cell count: changes with sustained weight loss. Obes Surg.

[10] Woo J, Yu R, You F (2013). Fitness, fatness and survival in elderly populations. Age (Dordr).

[11] Artero EG, España-Romero V, Jiménez-Pavón D, Martinez-Gómez D, Warnberg J, Gómez-Martínez S, González-Gross M, Vanhelst J, Kafatos A, Molnar D, De Henauw S, Moreno LA, Marcos A, Castillo MJ, HELENA study group (2014). Muscular fitness, fatness and inflammatory biomarkers in adolescents. Pediatr Obes.

[12] Magnussen CG, Schmidt MD, Dwyer T, Venn A (2012). Muscular fitness and clustered cardiovascular disease risk in Australian youth. Eur J Appl Physiol.

[13] Kim DJ, Noh JH, Lee BW, Choi YH, Jung JH, Min YK (2005). A white blood cell count in the normal concentration range is independently related to cardiorespiratory fitness in apparently healthy Korean men. Metabolism.

[14] Michishita R, Shono N, Inoue T, Tsuruta T, Node K (2008). Associations of monocytes, neutrophil count, and C-reactive protein with maximal oxygen uptake in overweight women. J Cardiol.

[15] Frohnert BI, Jacobs DR, Steinberger J, Moran A, Steffen LM, Sinaiko AR (2013). Relation between serum fatty acids and adiposity, insulin resistance, and cardiovascular risk factors from adolescence to adulthood. Diabetes.

[16] Tanner JM (1962). Growth at adolescence.

[17] Kuczmarski RJ, Oqden CL, Grummer-Strawn LM, Flegal KM, Guo SS, Wei R (2000). CDC growth charts: United States. Adv Data.

[18] Stofkova A (2009). Leptin and adiponectin: from energy and metabolic dysbalance to inflammation and autoimmunity. Endocr Regul.

[19] Iyer A, Fairlie DP, Prins JB, Hammock BD, Brown L (2010). Inflammatory lipid mediators in adipocyte function and obesity. Nat Rev Endocrinol.

[20] Zaldivar F, McMurray RG, Nemet D, Galassetti P, Mills PJ, Cooper DM (2006). Body fat and circulating leukocytes in children. Int J Obes.

[21] Kim JA, Park HS (2008). White blood cell count and fat distribution in female obese adolescents. Metabolism.

[22] Asadollahi K, Beeching NJ, Gill GV (2010). Leukocytosis as a predictor for non-infective mortality and morbidity. QJM.

[23] Brost SE (2004). The role of TNF- alpha in insulin resistance. Endocrine.

[24] Madjid M, Fatemi O (2013). Component of the complete blood count as risk predictors for coronary heart disease: in-depth review and update. Tex Heart Inst J.

[25] Ichihara Y, Hattori R, Anno T, Okuma K, Yokoi M, Mizuno Y (1996). Oxygen uptake and its relation to physical activity and other risk factors in asymptomatic middle-aged Japanese. J Cardiopulm Rehabil.

[26] Isasi CR, Deckelbaum RJ, Tracy RP, Starc TJ, Berglund L, Shea S (2003). Physical fitness an C- reactive protein level in children and young adults: the Columbia University BioMarkers Study. Pediatrics.

[27] Elosua R, Barteli B, Ordovas JM, Corsi AM, Lauretani F, Ferruci L, InCHIANT Investigators (2005). Association between physical activity, physical performance, and inflammatory biomarkers in an elderly population: the InCHIANTI study. J Gerontol A Biol Med Sci.

[28] Wärnberg J, Cunningham K, Romeo J, Marcos A (2010). Physical activity, exercise and low-grade systemic inflammation. Proc Nutr Soc.

[29] Blum CA, Müller B, Huber P, Kraenzlin M, Schindler C, De Geyter C (2005). Low-grade inflammation and estimates of insulin resistance during the menstrual cycle in lean and overweight women. J Clin Endocrinol Metab.

[30] Adamopoulos S, Parissis J, Kroupis C, Georgiadis M, Karatzas D, Karavolias G (2001). Physical training reduces peripheral markers of inflammation in patients with chronic heart failure. Eur Heart J.

