# Supplementary material for: Relation between leukocyte count, adiposity, and cardiorespiratory fitness in pubertal adolescents
Source: Einstein (Sao Paulo). 2014 Oct-Dec;12(4):420–4. doi: 10.1590/S1679-45082014AO3214 (PMC4879906; doi:10.1590/S1679-45082014AO3214)
Supplement: Translation

# Translation: Relação entre contagem de leucócitos, adiposidade e aptidão cardiorrespiratória em adolescentes púberes

**Authors:** Thiago Ricardo dos Santos Tenório, Breno Quintella Farah, Raphael Mendes Ritti-Dias, João Paulo Botero, Daniel Calado Brito, Patrícia Muniz Mendes Freire de Moura, Wagner Luiz do Prado

**Affiliations:** 1 Universidade de Pernambuco, Recife, PE, Brasil.; 2 Universidade Federal de São Paulo, Santos, SP, Brasil.

**Keywords:** Obesidade, Adolescente, Contagem de leucócitos, Adiposidade, Aptidão física, Inflamação

## Abstract

**Objetivo:**

Comparar a contagem total e diferencial de leucócitos de adolescentes obesos e eutróficos, e verificar suas possíveis relações com a aptidão cardiorrespiratória e indicadores de adiposidade.

**Métodos:**

Estudo transversal realizado com 139 adolescentes (107 obesos e 32 eutróficos), com idades entre 13 e 18 anos. A aptidão cardiorrespiratória foi determinada por análise direta de gases durante um teste incremental em esteira rolante. As contagens de leucócitos totais e subconjuntos foram estimadas por citometria de fluxo. A composição corporal foi avaliada pelo método de dupla energia de raios X. O teste *t* para amostras independentes foi utilizado para comparação entre os grupos. A relação entre leucócitos, aptidão cardiorrespiratória e indicadores de adiposidade foi verificada por meio dos testes de correlação de Pearson e regressão linear múltipla (ajustado para idade e índice de massa corporal).

**Resultados:**

Os adolescentes obesos apresentaram leucócitos (8,12±2,36u/L x 10^3^; p=0,001), neutrófilos (4,33±1,86u/L x 10^3^; p=0,002) e monócitos (0,70±0,22u/L x 10^3^; p=0,002) mais elevados em comparação com os níveis dos eutróficos. Após os ajustes necessários, a aptidão cardiorrespiratória foi negativamente associada com leucócitos, neutrófilos e monócitos em meninos.

**Conclusão:**

Adolescentes obesos apresentaram maior contagem de leucócitos totais e subpopulações, quando comparados aos eutróficos. Observou-se também uma fraca relação positiva entre adiposidade e leucócitos totais, monócitos e neutrófilos, e em meninos, uma relação negativa entre aptidão cardiorrespiratória e leucócitos totais, monócitos e neutrófilos.

## INTRODUÇÃO

A obesidade se tornou uma epidemia global, que afeta cerca de 500 milhões de adultos.^(1)^ A adiposidade excessiva está associada a diversos fatores de risco cardiometabólicos (por exemplo: intolerância à glicose, hipertrigliceridemia, hipercolesterolemia e aumento da pressão arterial sistólica),^(2)^ o que eleva a probabilidade de morte prematura.^(3)^ Além disso, evidências apontam que, na obesidade, existe um aumento importante da produção de marcadores inflamatórios e que grande parte dessas complicações cardiometabólicas estaria relacionada a esse estado inflamatório crônico sistêmico observado em indivíduos obesos.^(4,5)^

A contagem de leucócitos e subpopulações é um marcador clínico de processos inflamatórios^(6,7 ) ^relacionados a perturbações cardiometabólicas envolvidas no desenvolvimento de doenças cardiovasculares,^(8)^ especialmente em indivíduos com sobrepeso.^(9)^

Por outro lado, a aptidão cardiorrespiratória tem sido considerada um fator de proteção contra problemas de saúde, tanto em adultos^(10)^ quanto em crianças e adolescentes.^(11,12)^ Estudos anteriores demonstraram uma relação negativa independente entre níveis de leucócitos e aptidão cardiorrespiratória em homens^(13)^ e mulheres.^(14)^ No entanto, apesar de os distúrbios cardiometabólicos associados à obesidade poderem ser desencadeados ainda na primeira infância,^(15)^ pouco se sabe sobre essa associação na infância e na adolescência.

## OBJETIVO

Comparar a contagem total de leucócitos e seus subconjuntos em adolescentes obesos e eutróficos, além de investigar possíveis relações com a aptidão cardiorrespiratória e a adiposidade.

## MÉTODOS

Estudo transversal realizado na Escola Superior de Educação Física da Universidade de Pernambuco, com 139 adolescentes (15,21±1,51 anos), sendo 107 obesos (63 meninas) recrutados para o programa de tratamento da obesidade na adolescência da universidade e 32 adolescentes eutróficos (22 meninas). O estudo estendeu-se no período entre janeiro e abril de 2013.

Os adolescentes preencheram os seguintes critérios de inclusão: idade entre 13 e 18 anos e estágio maturacional entre 3 e 4, de acordo com os critérios de Tanner,^(16)^ a ausência de hipertensão e/ou outras doenças metabólicas (autorrelatadas ou identificadas por endocrinologista responsável), índice de massa corporal (IMC) acima do percentil 95 (obesos) e entre 25 e percentil 85 (eutróficos).^(17)^ Adolescentes que relataram uso excessivo de álcool, tabagismo, ou uso contínuo de anti-inflamatórios e/ou antialérgicos foram excluídos da amostra.

O estudo foi realizado de acordo com os princípios da Declaração de Helsinki, tendo sido formalmente aprovado pelo Comitê de Ética da Universidade de Pernambuco (CAAE: 15796113.9.0000.5207). O consentimento esclarecido dos pais ou responsável legal e o parecer favorável do participante foram obtidos depois de explicação detalhada dos procedimentos de todo protocolo do estudo.

### Antropometria e composição corporal

A estatura e a massa corporal foram medidas por meio de estadiômetro e balança de escala calibrada (Welmy^®^) com precisão de 0,1cm e 0,1kg, respectivamente. Na ocasião, os sujeitos posicionaram-se levemente vestidos e sem sapatos. O IMC foi calculado dividindo o peso corporal (kg) pela estatura elevada ao quadrado (m^2^). A composição corporal foi determinada pelo método de emissão de raios X de duplo feixe (DXA) (modelo QDR HOLOGIC WI).

### Aptidão cardiorrespiratória

O consumo de oxigênio pico (VO_2pico_) foi usado para determinar a aptidão cardiorrespiratória. VO_2_ foi analisado diretamente em um sistema metabólico respiratório de circuito aberto (Quark PFT, Cosmed, Itália) durante teste incremental contínuo em esteira rolante (Cosmed T200, Cosmed, Itália). Antes de cada teste, o equipamento foi calibrado para a composição do gás de referência (O_2_=12,2% e CO_2_=4,8%; White Martins), seguindo as recomendações do fabricante. A carga inicial foi fixada em 4km/h (aquecimento de 3 minutos) e elevada a 1km/h a cada minuto, com inclinação mantida constante a 1%. Os critérios de interrupção do teste foram fadiga volitiva, escala de Borg e relação de troca de gás superior a 18 e 1,15, respectivamente. O maior valor VO_2_ obtido antes da interrupção do teste foi considerado como o VO_2pico_.

### Análises sanguíneas

As amostras de sangue foram tomadas a partir da veia periférica do antebraço, com tubos contendo anticoagulante (EDTA), após jejum noturno (12 horas). A contagem total de leucócitos e de subpopulações (neutrófilos, monócitos e linfócitos) foi determinada por meio do método de citometria de fluxo fluorescente (Sysmex XE 2100^®^).

### Análise estatística

Para analisar a normalidade e a homogeneidade da distribuição dos dados, foram utilizados os testes Kolmogorov-Smirnorv e Levene, respectivamente. Para comparar a concentração de leucócitos e subconjuntos entre adolescentes obesos e eutróficos, foi utilizado o teste *t* de Student para amostras independentes ou teste de Mann-Whitney (não paramétrico). A correlação de *Spearman* foi utilizada para analisar a relação entre a composição corporal, aptidão cardiorrespiratória (VO_2pico_) e concentrações de leucócitos. Regressão linear múltipla ajustada para idade e IMC foi utilizada para analisar a relação independente entre aptidão cardiorrespiratória e contagem de leucócitos (e subpopulações). Todos os procedimentos estatísticos foram realizados utilizando o programa *Statistical Package for the Social Sciences* (SPSS Inc., Chicago, Estados Unidos), versão 20.0. O nível de significância estatística foi estabelecido em p<0,05.

## RESULTADOS

A tabela 1 mostra os parâmetros antropométricos, de composição corporal e aptidão cardiorrespiratória por grupos. Os adolescentes obesos apresentaram idade (p<0,001) e VO_2pico_ (p<0,001) menores; por outro lado, apresentaram IMC, percentual de gordura, massa gorda e massa magra (p<0,001, para todos) maiores em comparação com adolescentes eutróficos.

**Tabela 1:** Características antropométricas, de composição corporal e de aptidão cardiorrespiratória

Variáveis	Obesos (n=107)	Eutróficos (n=32)	Valor de p
**X±DP/MED(AI)**	**X±DP/MED(AI)**
Idade (anos)	14,28 (2,24)	16,78 (1,27)	0,001*
Massa corporal (kg)	93,73±13,51	56,32±7,30	0,001**
IMC (kg/m^2^)	34,28±4,06	20,79±1,86	0,001**
Gordura (%)	50,41±4,85	29,16±8,06	0,001**
Massa gorda (kg)	46,57 (13,12)	15,15 (12,50)	0,001*
Massa magra (kg)	44,26±7,27	38,20±8,16	0,001**
VO_2pico_ (ml.Kg/min^1^)	24,90±4,16	33,60±5,75	0,001**

X: média; DP: desvio padrão; MED: mediana; AI: amplitude interquartil; IMC: índice de massa corporal; VO_2pico_: consumo de oxigênio pico.*Teste U de Mann-Whitney; **teste *t* para amostras independentes.

Os adolescentes obesos apresentaram maiores contagens de leucócitos totais (8,12±2,36 x 10^3^u/L; p=0,036), neutrófilos (4,33±1,86 x 10^3^u/L; p=0,002) e monócitos (0,70±0 22 x 10^3^u/L; p=0,001) comparados com os eutróficos. Não houve diferença entre os grupos para a contagem da subpopulação de linfócitos (p=0,120) (Figura 1).

**Figura 1:** Comparação da contagem de leucócitos e subpopulações de adolescentes obesos e eutróficos. *p<0,05

Houve correlação significativa entre contagem total de leucócitos e o percentual de gordura (r=0,22), massa gorda − em quilograma (r=0,25), IMC (r=0,22) e VO_2pico_ (r=-0,22). Também foram observadas correlações significativas entre neutrófilos e percentual de gordura (r=0,27), massa gorda − em quilograma (r=0,24) e VO_2pico_ (r=-0,25); entre monócitos e massa gorda − em quilograma (r=0,19) e IMC (r=0,18); e entre a razão neutrófilos/linfócitos e percentual de gordura (r=0,22) e a massa gorda − em quilograma (r=0,18) (Tabela 2).

**Tabela 2:** Correlação entre contagem de leucócitos e subconjuntos com parâmetros antropométricos, de composição corporal e aptidão cardiorrespiratória (n=139)

Variáveis	%G	MG	MM	IMC	VO_2pico_
Leucócitos totais (u/L x 10^3^)	0,27*	0,25*	0,08	0,22*	-0,22*
Neutrófilos (u/L x 10^3^)	0,27*	0,24*	0,05	0,21*	-0,25*
Monócitos (u/L x 10^3^)	0,17	0,19*	0,13	0,18*	-0,10
Linfócitos (u/L x 10^3^)	0,13	0,15	0,11	0,12	-0,03
Razão neutrófilos/linfócitos	0,22*	0,18*	-0,01	0,17	-0,24

%G: porcentual de gordura; MG: massa gorda; MM: massa magra; IMC: índice de massa corporal; VO_2pico: _consumo de oxigênio pico. Correlação de *Spearman* *p< 0,05.

A tabela 3 demonstra as relações entre leucócitos totais, neutrófilos e monócitos com a aptidão cardiorrespiratória, quando realizados ajustes para idade e IMC. Nos meninos, leucócitos totais, neutrófilos e monócitos se mostraram negativamente associados com a aptidão cardiorrespiratória. Não houve esse tipo de relação entre as meninas.

**Tabela 3:** Associação entre aptidão cardiorrespiratória, leucócitos totais, neutrófilos e monócitos (n=139)

Variáveis dependentes	Aptidão cardiorrespiratória (VO_2pico_)		
	β	EP	Valor de p
Meninos			
Leucócitos totais (u/Lx10^3^)	-0,534	0,095	0,013
Neutrófilos (u/Lx10^3^)	-0,545	0,067	0,012
Monócitos (u/Lx10^3^)	-0,019	0,008	0,042
Meninas			
Leucócitos totais (u/Lx10^3^)	-0,003	0,093	0,977
Neutrófilos (u/Lx10^3^)	0,013	0,078	0,868
Monócitos (u/Lx10^3^)	-0,002	0,008	0,998

Regressão linear múltipla: modelo ajustado para idade e índice de massa corporal. β: coeficiente de regressão; EP: erro padrão.

## DISCUSSÃO

Os principais achados do presente estudo foram os seguintes: adolescentes obesos apresentaram maior contagem de leucócitos totais e subpopulações (neutrófilos e monócitos) quando comparados aos pares eutróficos; houve relação positiva entre adiposidade e leucócitos totais, monócitos e neutrófilos; houve também relação negativa entre aptidão cardiorrespiratória e leucócitos totais, monócitos e neutrófilos, somente nos meninos, independentemente do IMC e idade.

Estudos anteriores relataram um estado de inflamação crônica e de baixo grau em adolescentes obesos.^(18,19)^ As contagens de leucócitos mais elevadas observadas em adolescentes obesos no presente estudo foram semelhantes aos resultados em adultos,^(9)^ em crianças^(20)^ e adolescentes.^(21)^ De fato, as correlações positivas encontradas entre a contagem total de leucócitos e subpopulações (neutrófilos e monócitos), mesmo de intensidades fracas (r=0,18 a r=0,27), já demonstradas em estudos anteriores,^(6,7)^ evidenciaram que, em parte, a contagem de leucócitos totais, monócitos e neutrófilo sofre influência dos indicadores de adiposidade, com reflexo, de forma global, no perfil inflamatório do adolescente.

Outro ponto importante diz respeito à diferença significativa entre as idades dos adolescentes obesos e eutróficos. No entanto, todos os adolescentes obtiveram a classificação do estado nutricional por meio de curvas de percentis^(17)^ que levam em consideração a idade individual de cada adolescente. Adicionalmente, as análises de regressão, foram todas corrigidas pela idade, a fim de isolar qualquer influência dessa variável sobre os resultados encontrados.

Em adultos, tem sido descrito que alta contagem de leucócitos é um fator de risco independente para o desenvolvimento de doenças cardiovasculares.^(22)^ O mecanismo para esse efeito não está totalmente esclarecido, mas está provavelmente relacionado com a liberação de radicais livres, moléculas pró-coagulantes e enzimas proteolíticas por neutrófilos e monócitos, o que pode acelerar o processo de formação de placa aterosclerótica.^(20)^ Além disso, os monócitos secretam fator de necrose tumoral alfa (TNF-α), uma citocina que se relaciona com a resistência à insulina.^(23)^

Adicionalmente, neutrófilos e monócitos têm sido associados com doença arterial coronariana.^(24)^ Nossos resultados sugerem que os efeitos desses processos prejudiciais podem se iniciar cada vez mais cedo, o que, por sua vez, pode indicar que a obesidade na adolescência pode desencadear o aparecimento da doença arterial coronariana.

Outro achado importante do presente estudo foi uma correlação negativa entre o VO_2pico_ e a contagem de leucócitos totais e neutrófilos. Embora o VO_2pico_ seja considerado um marcador de aptidão física, também é importante destacar que tal parâmetro reflete a função do sistema cardiopulmonar, sendo considerado um parâmetro de proteção contra os fatores de risco para aterosclerose.^(25 ) ^Michishita et al.,^(14)^ em um estudo com mulheres com excesso de peso, observaram correlação negativa entre monócitos e VO_2pico_. Dessa maneira acredita-se que a elevação na aptidão cardiorrespiratória de adolescentes obesos pode influenciar na diminuição da produção de marcadores inflamatórios, proporcionando um maior estafo anti-inflamatório, que é importante na prevenção de eventos cardiovasculares.

Interessantemente, a associação entre aptidão cardiorrespiratória e contagem de leucócitos e subpopulações foi observada apenas em meninos, após ajuste para o IMC e idade. Tal resultado está de acordo com o de estudos anteriores em populações de idades mais avançadas.^(26,^27^)^ A razão para essa diferença entre os gêneros não está totalmente compreendida, mas o nível de atividade física mais baixo, geralmente observados em meninas, é um potencial fator.^(28)^ Além disso, há uma estreita relação entre esteroides sexuais, inflamação e distribuição de gordura corporal em mulheres, pois, devido ao ciclo menstrual, os níveis circulantes de marcadores inflamatórios podem variar significativamente.^(29)^

Nesse sentido, a aderência de leucócitos, principalmente os monócitos e neutrófilos, ao endotélio, e também a migração dessas células para a parede do vaso sanguíneo são características envolvidas em várias fases da aterosclerose.^(6)^ Adamopoulos et al.^(30)^ demonstraram que o aumento do VO_2pico_ após período de treinamento aeróbio foi eficaz para inibir a infiltração de monócitos na parede do vaso. Assim, níveis mais elevados de VO_2pico_ proporcionam efeitos cardiovasculares protetores, inibindo processos inflamatórios.

As principais limitações do estudo foram a análise isolada da contagem de leucócitos, uma vez que a avaliação de sua função e de sua ativação poderia fornecer informações importantes; o não controle dos períodos menstruais das adolescentes; e, finalmente, a não avaliação da influência do padrão de distribuição de gordura.

Por outro lado, vale salientar que, no presente estudo, o VO_2pico_ foi determinado por análise direta de gases, e a composição corporal foi avaliada por meio da técnica de absortometria de duplo feixe (DXA). Tais métodos reforçam os resultados deste estudo, por se tratarem de técnicas mais precisas e de maior acurácia, principalmente em relação à população em estudo.

## CONCLUSÃO

Níveis mais elevados de leucócitos (monócitos e neutrófilos) foram observados em adolescentes obesos em comparação com os eutróficos. Esse perfil sugeriu um estado pró-inflamatório crônico nesses adolescentes obesos, o que poderia estar relacionado com a adiposidade excessiva e à aptidão cardiorrespiratória diminuída. Tais dados destacam a necessidade eminente para o desenvolvimento de intervenções nessa população visando não somente ao controle de peso, mas também a melhorias na aptidão cardiorrespiratória.
